# Epithelial-to-mesenchymal transition drives cancer genomic instability

**DOI:** 10.1186/s13046-025-03402-x

**Published:** 2025-04-30

**Authors:** Julienne L. Carstens, Sara Lovisa

**Affiliations:** 1 Department of Medicine, Division of Hematology & Oncology, University of Alabama at Birmingham, Birmingham, AL USA; 2 O’Neal Comprehensive Cancer Center, University of Alabama at Birmingham, Birmingham, AL USA; 3 Immunology Institute, University of Alabama at Birmingham, Birmingham, AL USA; 4 Department of Biomedical Sciences, Humanitas University, 20072 Pieve Emanuele, Milan, Italy; 5 Department of Gastroenterology, IRCCS Humanitas Research Hospital, 20089 Rozzano, Milan, Italy

**Keywords:** EMT, Epithelial-to-mesenchymal transition, Genomic instability, Cell plasticity, Cancer

## Abstract

Epithelial-to-Mesenchymal Transition (EMT) is a form of embryonic cell plasticity reactivated in adult cells during injury and cancer. A recent study by Perelli et al. demonstrates that EMT confers an evolutionary advantage to tumors by inducing chromosomal instability, structural genomic rearrangements and chromothripsis, thus favoring the emergence of high-fitness malignant clones.

## Background

Epithelial-to-Mesenchymal Transition (EMT) is a form of cell plasticity occurring during embryonic development that enables the primordial epithelium to transdifferentiate into cells with migratory capacity and form a second embryonic layer termed mesoderm. This process is usually silent in adult tissues but reactivates concomitantly with pathological situations disrupting epithelial homeostasis, such as injury and neoplastic transformation [1]. Phenotypically EMT causes the loss of the epithelial identity and the acquisition of morphological features of mesenchymal cells, a process that is orchestrated at both transcriptional and translational levels. The phenotypic change is accompanied by novel functional aspects mainly related to promoting chronic tissue injury, chemoresistance and metastasis, although this latter is still a matter of discussion. While in these past decades EMT has been widely investigated for its involvement in the metastatic cascade, the functional consequences of its activation in the milieu of the primary tumor have been understudied.

## Main text

A recently released study [2] employed new sophisticated mouse models and multiple single-cell and spatial omics to demonstrate the leading role of EMT in driving cancer evolution by the generation of a genomic entropy, advantageous for the growth of high fitness cancer lineages. In this study, the authors generated elegant somatic mosaic genetically-engineered mouse models (SM-GEMM) to specifically trace and perturb EMT cells as defined by the expression of the mesenchymal marker vimentin. They employed a SM-GEMM model of pancreatic adenocarcinoma (PDAC) obtained by the orthotopic injection of adeno-associated viruses carrying Cre recombinase and sgRNAs against Tp53 and/or Cdkn2a/b in mice carrying LSL-KRas^G12D^, H11-LSL-Cas9 and R26-LSL-FSF-tdTomato conditional alleles. These mice were further crossed with a newly generated allele obtained by engineering the mouse *Vimentin* locus with a conditional Flex-based construct carrying GFP and Flippase. In the obtained quadruple model (named PCФ), three layers of regulation are guaranteed. First, intraductal virus injection leads to pancreatic adenocarcinoma development. Second, the delivered Cre also recombines the flexed construct thus allowing GFP and Flippase expression when *Vimentin* is transcribed. Third, the coordinated action of Cre and Flippase permits the tdTomato^+^ lineage tracing of cancer cells which express vimentin, thus fate mapping EMT cells. Therefore the integrated use of orthogonal recombinases, GFP reporter and tdTomato lineage tracer allows the identification of three cellular states: cells actively in EMT (GFP^high^Tom^+^) and EMT-derived progeny (GFP^low^Tom^+^) collectively referred to as mesenchymal lineages (MS-L), and epithelial cells (GFP^–^Tom^–^, termed EPI-L). In these PCФ mice, advanced pancreatic tumors and metastasis were almost universally established by the mesenchymal lineages, regardless of driver mutation combinations and with a high proportion of cells still displaying active EMT state. The metastatic lesions however displayed various site-specific ratios between active EMT and EMT-derived progeny. This observation was confirmed by orthotopic transplantation of tdTom^−^ and tdTom^+^ organoids. Interestingly, tdTom^−^ transplants were able to originate tdTom^+^ lesions, thus indicating that epithelial cells are prone to mesenchymal reprograming.

The construction of a second mouse model (PCΨ) for ablating EMT-proficient cells early in the tumorigenesis by leveraging a diphtheria toxin suicide cassette (DTA) demonstrated that EMT depletion completely abrogates tumor development, progression, and metastasis. In fact, the ablated mice only developed low-grade cysts lesions with low proliferative index. The authors also developed a third model to ablate the actively proliferating EMT lineage by knocking-in the *Vimentin* locus with a conditional Flex-based construct carrying GFP and the viral timidine kinase (TK), which kills proliferating cells upon ganciclovir (GCV) administration (PCΩ model). In these mice, GCV administration leads to the selective ablation of proliferating, vimentin-expressing EMT cells. Two cellular states are identifiable in this model: epithelial cells (Tom^+^GFP^−^, termed EPI-L) and cells actively in EMT state (Tom^+^GFP^+^, termed MS-L). Results show that GCV delivery completely abrogated primary tumor and metastasis growth when administered both in a short (21 days) and a long (90 days) treatment regimen. Pulse-chasing GCV in both treatment schedules led to the development of tumors and metastasis similarly to the vehicle control. In line with the transplant model, this result indicates the intrinsic capacity of the epithelium to activate a mesenchymal reprograming. Moreover, this model demonstrates the prominent contribution of EMT to the proliferating tumor compartment. Histopathological analysis of the PCΩ tumors confirmed that MS-L cells display higher proliferation index, higher fraction of cells in G2/M phase as marked by pHH3, and higher number of aberrant mitosis.

Spatial profiling by Slide-DNA-seq of the genomic landscape of early PCФ tumors revealed chromosomal instability (CIN) in the areas enriched for EMT-derived mesenchymal cells. Genomic instability was further investigated by Whole Genome Sequencing (WGS) on PCΨ and PCΩ tumors where the EMT-derived populations have been ablated or not. Impeding EMT in the ablated tumors resulted in reduced copy number alterations, whole genome duplication and number of structural variants therefore abolishing the high genomic instability present in the EMT proficient tumors. A similar flattening of the genomic arrangements was observed in pancreatic tumors derived from the KPCY; Zeb1^cKO^ model in which the deletion of the EMT-transcription factor (TF) Zeb1 reduces EMT and tumor progression. This result further reinforces the discovery of the aberrant genomic alterations driven by EMT.

A copy number signature particularly depleted in the EMT deficient tumors was the one relative to chromotriptic events, which consist in massive rearrangements at a certain chromosomal location. In fact, tumors with enabled mesenchymal plasticity presented a high burden of chromotripsis. This phenomenon appears to be conserved across species and tissues as interrogation of multiple patient databases (TCGA, COMPASS and MDA-PDX-PDAC) highlighted that epithelial tumors of different origin presenting mesenchymal reprograming concordantly display chromotriptic events and genomic rearrangements. Additionally, the structural rearrangements repeatedly observed in chromosomes such as ch6 regards chromotriptic events occurring in the *KRas* locus. A marked KRas allelic imbalance in fact emerged in the EMT proficient tumors, thus suggesting a role for EMT in directly shaping the genomic landscape in favor of advantageous driver events.

Further into the molecular aspects of EMT plasticity, scRNAseq and scGET-seq performed on short-term culture of PCΩ EPI-L and MS-L cells confirmed the enrichment for structural variants in the mesenchymal lineage, occurring particularly in centromeric and peri-centromeric chromosomal regions which may be responsible for the delayed mitosis and CIN, and permissive of catastrophic genomic events like chromotripsis. Accordingly, MS-L cells display enlarged nuclei, prolonged mitosis duration, increased number of chromosomes and increased DNA damage pathway. All these events are reflective of profound nuclear and mitotic abnormalities which seed the ground for subsequent genomic alterations, as well documented by the in vivo data. By computational inference, the authors demonstrated that intratumor transcriptional heterogeneity is highly promoted by EMT and that this mesenchymal plasticity empowers cancer cells favoring the growth of high fitness clones.

**Fig. 1:**
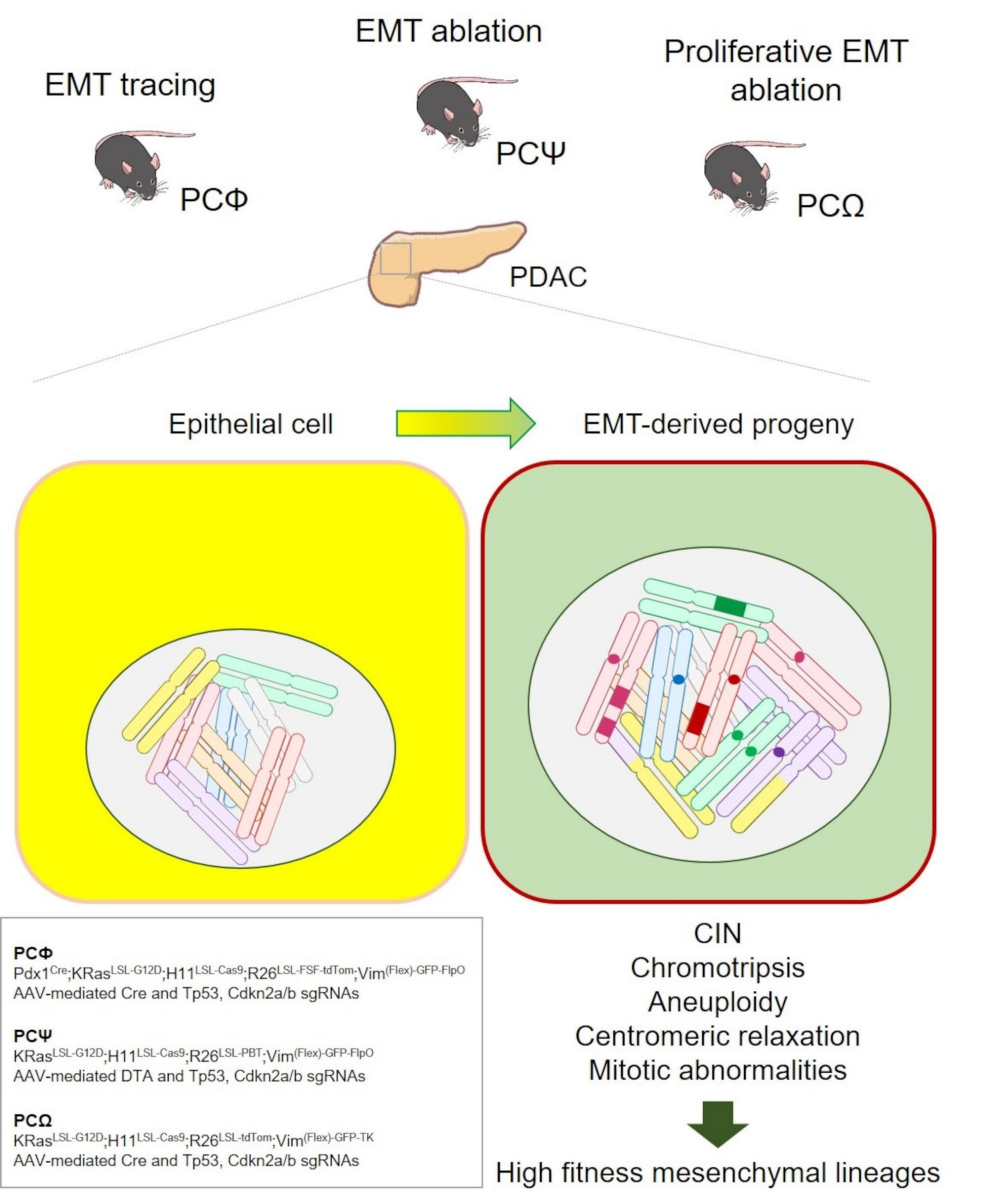
REvolutionary EMT promotes genomic instability and cancer progression. By utilizing multiple different genetic approaches, Perelli *et al*. demonstrated that EMT activation in tumor cells induces chromosomal instability and profound genomic alterations such as chromotripsis. Moreover, EMT is associated with increased chromatin accessibility at centromeric and pericentromeric regions resulting in delayed mitosis and catastrophic cell division. EMT: Epithelial-to-Mesenchymal Transition; PDAC: pancreatic adenocarcinoma. CIN: chromosomal instability. Figure was prepared utilizing illustrations from NIAID NIH BIOART Source

This study provides for the first time compelling evidences on the functional involvement of EMT in shaping tumor evolution and unequivocally demonstrates the genomic implications of epithelial plasticity (Fig. 1). Although it does not address the molecular mechanisms by which EMT induces genomic instability, previous studies provided some evidences in this regard. In fact, it was shown that EMT cells overriding TGFβ-induced proliferation arrest present aneuploidy caused by cytokinesis failure due to suppression of multiple nuclear envelope proteins [3]. The inhibition of the EMT-TF Zeb1 rescued these defects [3]. Abnormal G2/M checkpoint leading to defects in chromosomal segregation and cytokinesis was also observed in a skin model upon Snail expression [4]. The overexpression of another EMT-TFs, namely Twist1, induces nuclear and mitotic aberration in colorectal cancer by downregulating factors involved in cell cycle checkpoints preserving genomic stability [5]. In the same cancer context, however, Zeb2 overexpressing tumors do not present such genomic alterations, possibly indicating heterogeneity in the EMT programs [6].

## Conclusions

The genetic approach utilized by Perelli and co-workers, based on the combined use of orthogonal recombinases, flexed constructs and dual fluorescence, collectively centered around the activation of the *Vimentin* locus, represents a substantial methodological advancement for the study of EMT compared to the widely employed mouse models to manipulate the EMT-TFs. Although defining EMT only based on the phenotypic acquisition of vimentin expression may be too simplistic, it certainly covers the mesenchymal plasticity in its more extensive aspects, independently from the possible heterogeneity of each individual TFs-induced programs. The epithelial-confined perturbation of EMT obtained with these generated models removes the risk of a confounding contribution of the microenvironmental components sharing the vimentin positivity.

Certainly this study opens up multiple interesting questions. The first one regards where does EMT stand with respect to the temporal events leading to the reported genomic abnormalities? Scattered presence of EMT-derived cells was detected in pre-neoplastic lesions, thus highlighting a very early activation of the mesenchymal plasticity, as also previously reported [7]. Additionally, the most prominent genomics rearrangements have been observed in the PCΨ model targeting early tumorigenesis, further suggesting EMT as an early event occurring during neoplastic transformation. Consequently, if EMT has such an early onset, how is it temporally positioned with regards to oncogene activation? Does EMT drive the oncogenes or vice-versa? Previous studies have indicated a synergistic cooperation between EMT-TFs and various oncogenes, but a temporal reconstruction of what comes first has never been dissected. Here EMT-induced chromotriptic events on chromosome 6 were shown to produce copy number amplifications of oncogenic drivers including *KRas*. This implies EMT plasticity as an early neoplastic event favoring the accumulation of key genetic events. Future work with an additional level of sophistication including temporally controlled genetics will provide further insights into the timeline of these events.

Another aspect of the biology of EMT has emerged thanks to the use of the TK suicide system. Based on the results obtained with this model, the vimentin^+^ EMT cells are highly proliferative cells and their ablation inhibits tumor growth at primary and metastatic sites. This seems to be counterintuitive with the general idea that adult EMT cells exhibit low proliferation [8–11] though during embryogenesis motility and proliferation are concomitant features of the endoderm undergoing EMT and have been associated with the most aggressive mesenchymal cancer types [12]. It is also possible that heterogeneity exists in the proliferative capacity, with different degrees along with the mesenchymal continuum. The model presented in this study captures the highly mesenchymal/sarcomatoid and proliferative version, and may not fully recapitulate alternative modes of partial-EMT that have been reported [13, 14]. Moreover, the plasticity between the different cell-states of the EMT continuum as well as the influence of these heterogeneous populations have on the others’ proliferation [15, 16] may not be entirely modelled by the available reporter systems.

Besides the highlighted limitations, this work definitely provides significant advancement and novelty in our comprehension of the processes of epithelial plasticity and EMT.

## Data Availability

No datasets were generated or analysed during the current study.
